# DNA-hydrolyzing catalytic IgGs from schizophrenia patients do not affect cell viability of the SH-SY5Y human neuroblastoma cell line

**DOI:** 10.1192/j.eurpsy.2022.2002

**Published:** 2022-09-01

**Authors:** E. Epimakhova, E. Ermakov, D. Kazantseva, D. Parshukova, E. Dmitrieva, L. Smirnova, S. Ivanova, A. Semke

**Affiliations:** 1 Tomsk National Research Medical Center of the Russian Academy of Sciences, Mental Health Research Institute, Tomsk, Russian Federation; 2 Institute of Chemical Biology and Fundamental Medicine, Laboratory Of Repair Enzymes, Novosibirsk, Russian Federation; 3 Siberian State Medical University, Department Of Biomedicine, Tomsk, Russian Federation; 4 Mental Health Research Institute, Tomsk National Research Medical Center of the Russian Academy of Sciences, Department Of Endogenous Disorders, Tomsk, Russian Federation

**Keywords:** neuroblastoma cell line, schizophrenia, DNA-hydrolyzing activity

## Abstract

**Introduction:**

DNA-hydrolyzing catalytic IgGs have caspase-dependent cytotoxic effects in autoimmune diseases. Recently, DNA-hydrolyzing IgGs have been discovered in schizophrenia. However, their cytotoxic properties have not been studied.

**Objectives:**

To assess the effect of serum IgGs with DNA-hydrolyzing activity of schizophrenia patients on the cell viability of the SH-SY5Y human neuroblastoma cell line.

**Methods:**

Serum of 8 patients with paranoid schizophrenia in the acute phase and 7 mentally and somatically healthy persons were used. IgG was purified from serum by affinity chromatography on Protein-G-Sepharose columns. The DNA hydrolyzing activity of IgG was assessed by the degree of hydrolysis of the pBluescript plasmid. The cell viability of the SH-SY5Y human neuroblastoma cell line after exposure to purified IgG preparations was assessed by high-throughput screening on the CellInsight CX7 platform (Thermo Scientific, USA) using the fluorescent dyes propidium iodide and Hoechst.

**Results:**

Of the 8 IgG preparation obtained, 4 drugs had high DNA-hydrolyzing activity. All tested IgG preparations from healthy donors were inactive. One-way ANOVA analysis of the proportion of dead cells of the SH-SY5Y line after exposure to antibodies (0.1 mg/ml) showed no significant differences in the proportion of dead cells (p=0.688 after 24 hours; p=0.831 after 48 hours). Similar results were obtained at a higher concentration of antibodies - 0.2 mg/ml.

**Conclusions:**

Thus, it has been shown *in vitro* that IgGs isolated from the serum of schizophrenia patients with or without DNA-hydrolyzing activity does not exhibit cytotoxic properties against the SH-SY5Y human neuroblastoma cell line. *Support by Grant of RSF № 18-15-00053P.*

**Disclosure:**

No significant relationships.

